# Effect of Combined Immune Checkpoint Inhibition vs Best Supportive Care Alone in Patients With Advanced Colorectal Cancer: The Canadian Cancer Trials Group CO.26 Study

**DOI:** 10.1001/jamaoncol.2020.0910

**Published:** 2020-05-07

**Authors:** Eric X. Chen, Derek J. Jonker, Jonathan M. Loree, Hagen F. Kennecke, Scott R. Berry, Felix Couture, Chaudhary E. Ahmad, John R. Goffin, Petr Kavan, Mohammed Harb, Bruce Colwell, Setareh Samimi, Benoit Samson, Tahir Abbas, Nathalie Aucoin, Francine Aubin, Sheryl L. Koski, Alice C. Wei, Nadine M. Magoski, Dongsheng Tu, Chris J. O’Callaghan

**Affiliations:** 1 Princess Margaret Cancer Center, Toronto, Canada; 2 The Ottawa Hospital, Ottawa, Canada; 3 BC Cancer, Vancouver, Canada; 4 Virginia Mason Medical Center, Seattle; 5 Department of Oncology, Queen’s University, Kingston, Canada; 6 CHU de Québec-Université, Laval, Canada; 7 Eastern Health, St John’s, Canada; 8 Juvravinski Cancer Center, Hamilton, Canada; 9 Segal Cancer Center, Montreal, Canada; 10 Moncton Hospital, Moncton, Canada; 11 Dalhousie University, Halifax, Canada; 12 Hôpital Sacré-Coeur de Montréal, Montreal, Canada; 13 Sherbrooke University, Sherbrooke, Canada; 14 Saskatoon Cancer Center, Saskatoon, Canada; 15 Hôpital Cité-de-la-Santé, Laval, Canada; 16 Centre de recherche du Centre hospitalier de l'Université de Montréal (CHUM), Montreal, Canada; 17 Cross Cancer Center, Edmonton, Canada; 18 Canadian Cancer Trials Group, Kingston, Canada

## Abstract

**Importance:**

Single-agent immune checkpoint inhibition has not shown activities in advanced refractory colorectal cancer (CRC), other than in those patients who are microsatellite-instability high (MSI-H).

**Objective:**

To evaluate whether combining programmed death-ligand 1 (PD-L1) and cytotoxic T-lymphocyte–associated protein 4 (CTLA-4) inhibition improved patient survival in metastatic refractory CRC.

**Design, Setting, and Participants:**

A randomized phase 2 study was conducted in 27 cancer centers across Canada between August 2016 and June 2017, and data were analyzed on October 18, 2018. Eligible patients had histologically confirmed adenocarcinoma of the colon or rectum; received all available standard systemic therapies (fluoropyrimidines, oxaliplatin, irinotecan, and bevacizumab if appropriate; cetuximab or panitumumab if *RAS* wild-type tumors; regorafenib if available); were aged 18 years or older; had adequate organ function; had Eastern Cooperative Oncology Group performance status of 0 or 1, and measurable disease.

**Interventions:**

We randomly assigned patients to receive either 75 mg of tremelimumab every 28 days for the first 4 cycles plus 1500 mg durvalumab every 28 days, or best supportive care alone (BSC) in a 2:1 ratio.

**Main Outcomes and Measures:**

The primary end point was overall survival (OS) and a 2-sided P<.10 was considered statistically significant. Circulating cell-free DNA from baseline plasma was used to determine microsatellite instability (MSI) and tumor mutation burden (TMB).

**Results:**

Of 180 patients enrolled (121 men [67.2%] and 59 women [32.8%]; median [range] age, 65 [36-87] years), 179 were treated. With a median follow-up of 15.2 months, the median OS was 6.6 months for durvalumab and tremelimumab and 4.1 months for BSC (hazard ratio [HR], 0.72; 90% CI, 0.54-0.97; *P* = .07). Progression-free survival was 1.8 months and 1.9 months respectively (HR, 1.01; 90% CI, 0.76-1.34). Grade 3 or 4 adverse events were significantly more frequent with immunotherapy (75 [64%] patients in the treatment group had at least 1 grade 3 or higher adverse event vs 12 [20%] in the BSC group). Circulating cell-free DNA analysis was successful in 168 of 169 patients with available samples. In patients who were microsatellite stable (MSS), OS was significantly improved with durvalumab and tremelimumab (HR, 0.66; 90% CI, 0.49-0.89; *P* = .02). Patients who were MSS with plasma TMB of 28 variants per megabase or more (21% of MSS patients) had the greatest OS benefit (HR, 0.34; 90% CI, 0.18-0.63; *P* = .004).

**Conclusions and Relevance:**

This phase 2 study suggests that combined immune checkpoint inhibition with durvalumab plus tremelimumab may be associated with prolonged OS in patients with advanced refractory CRC. Elevated plasma TMB may select patients most likely to benefit from durvalumab and tremelimumab. Further confirmation studies are warranted.

**Trial Registration:**

ClinicalTrials.gov Identifier: NCT02870920

## Key Points

**Question:**

Can combined immune checkpoint inhibition improve overall survival (OS) in patients with advanced refractory colorectal cancer (CRC)?

**Findings:**

In this randomized phase 2 study with 180 patients randomized in a 2:1 ratio to tremelimumab and durvalumab plus best supportive care or best supportive care alone, the median OS was 6.6 months for durvalumab and tremelimumab and 4.1 months for best supportive care; correlative analysis revealed that patients with plasma tumor mutation burden (TMB) of 28 or more variants per megabase had the greatest OS benefit.

**Meaning:**

Combined immune checkpoint inhibition may prolong OS in patients with advanced refractory CRC.

## Teaser

This phase 2 randomized clinical trial evaluates the outcomes of combined immune checkpoint inhibition plus best supportive care vs best supportive care alone for patients with advanced colorectal cancer.

## Introduction

Colorectal cancer is the second leading cause of cancer death worldwide, accounting for approximately 880 000 deaths in 2018.^1^ According to the American Cancer Society, there are expected to be 51 020 deaths from advanced colorectal cancer in 2019 in the United States.^2^ For most patients with advanced colorectal cancer, systemic therapy is the main treatment modality with median overall survival (OS) approaching 30 months in clinical trials.^3,4^

Despite recent approval of agents such as trifluridine/tipiracil (TAS-102) and regorafenib, outcomes for patients with advanced colorectal cancer remain poor and new treatments are needed.^5,6^ Monoclonal antibodies (mAb) against programmed death receptor-1 (PD-1), pembrolizumab and nivolumab, have shown considerable activity in patients with advanced colorectal cancer with DNA mismatch repair deficient/microsatellite instability-high (dMMR/MSI-H) tumors.^7,8,9^ However, single-agent immune checkpoint inhibitors have not shown meaningful activities in DNA mismatch repair proficient/microsatellite stable (pMMR/MSS) colorectal cancer. Recently, combined blockade with nivolumab and the cytotoxic T-cell lymphocyte antigen-4 (CTLA-4) antibody, ipilimumab, demonstrated additional benefit compared with nivolumab alone in patients with dMMR/MSI-H tumors.^10^

Durvalumab (Imfinzi, AstraZeneca) is a selective, high-affinity human IgG1 mAb against programmed death ligand 1 (PD-L1), whereas tremelimumab is a selective human IgG2 mAb against CTLA-4. Durvalumab and tremelimumab can be combined with manageable adverse events.^11^ We hypothesized that a combined blockade of PD-L1 and CTLA-4 would provide greater anticancer activity and conducted a randomized phase 2 clinical trial to evaluate the efficacy of durvalumab and tremelimumab in patients with advanced refractory colorectal cancer.

## Methods

### Patients

The trial protocol is available in Supplement 1. Eligible patients (121 men [67.2%] and 59 women [32.8%]; median [range] age, 65 [36-87] years) gave written informed consent; had histologically confirmed adenocarcinoma of the colon or rectum; received all available standard systemic therapies (fluoropyrimidines, oxaliplatin, irinotecan, and bevacizumab if appropriate; cetuximab or panitumumab if *RAS* wild-type; regorafenib if available); were aged 18 years or older; had adequate hematologic, renal, and liver function; had Eastern Cooperative Oncology Group (ECOG) performance status of 0 or 1, and measurable disease according to Response Evaluation Criteria in Solid Tumors (RECIST, version 1.1).^12^ Patients were excluded if they received prior mAbs targeting PD-1, PD-L1, or CTLA-4, or had a history of autoimmune disorders or severe immune-mediated toxic effects.

The study was approved by the institutional review board of each participating center, conducted according to the principles of the Declaration of Helsinki, complied with all applicable regulations, and was registered on ClinicalTrials.gov (NCT02870920).

### Randomization

Patients were randomized, in a 2:1 ratio, to receive 75 mg of tremelimumab intravenously every 4 weeks for the initial 4 cycles only, durvalumab 1500 mg of intravenously every 4 weeks, and best supportive care (BSC) (the treatment group) or BSC alone. The randomization was dynamically balanced by ECOG performance status (0 or 1), and the site of primary tumor using the method of minimization. Randomization was performed centrally by the Canadian Cancer Trials Group (CCTG) central office. The study was open label, and investigators and patients were not blinded to treatment assignments. No crossover was allowed between treatment groups.

### Study Assessments

Patients were evaluated clinically every 4 weeks while on study treatments, and every 12 weeks after disease progression. Radiological assessments with computed tomographic images were performed every 8 weeks until progression. Treatments continued until there was radiological or clinical evidence of disease progression, intolerable toxic effects, withdrawal of consent, or death. Adverse events were collected and classified according to the National Cancer Institute Common Toxicity Criteria for Adverse Events, version 4.0.^13^

Blood samples for circulating cell-free DNA (cfDNA) were collected prior to study therapy, at 8 weeks, and at the time of disease progression. Baseline samples were analyzed using the GuardantOMNI next generation sequencing 2.15 Mb, 500-gene panel (Guardant Health, Inc) to identify single nucleotide variants (SNVs), indels, fusions, copy number amplifications, MSI-high status, and tumor mutation burden (TMB).^14^ Plasma TMB was reported as variations per megabase (vts/Mb) by the GuardantOMNI algorithm, which includes all somatic synonymous and nonsynonymous SNVs and indels excluding germline, clonal hematopoiesis of indeterminate potential (CHIP), driver and resistance variations with statistical adjustment for sample-specific tumor shedding and molecular coverage. Validation of plasma TMB and MSI have been previously described.^15,16^

Quality of life was assessed using EORTC QLQ-C30 at baseline, 4, 8, 12, 16, 24 weeks, then every 12 weeks until deterioration to ECOG PS 4 or death.^17^

### Statistical Analysis

The primary end point was OS, defined as the time from randomization to death from any cause. Secondary end points included progression-free survival ([PFS], the time from randomization to the first objective documentation of disease progression or death from any cause), objective response rate ([ORR], the proportion of patients with a documented complete response [CR] or partial response [PR]), toxic effects, and safety.

This study was designed to observe 150 deaths to have a power of 80% and a 2-sided α of 10% to detect a 35% reduction in the continuous risk of death. This assumption translated to a hazard ratio (HR) of 0.65, and corresponded to an increase of the median OS from 4.5 months for BSC to 6.9 months for the treatment group. It was calculated that 180 patients had to be enrolled over 18 months and followed for 6 months to observe the required number of deaths.

Overall survival and PFS were analyzed according to intention-to-treat. They were summarized by Kaplan-Meier method, and compared by a stratified log-rank test adjusting for ECOG performance status and site of the primary tumor. The HRs and 90% CIs were calculated based on a stratified Cox proportional hazard model. A Cochran-Mantel-Haenszel test was used to compare ORR between the 2 study groups adjusting for ECOG Performance Status and site of the primary tumor. Adverse events were analyzed according to treatment received and compared by Fisher exact tests among patients who received at least 1 dose of protocol treatments. Data were analyzed on October 18, 2018, and SAS statistical software (version 9.0; SAS Institute, Inc) was used for analysis.

In an exploratory analysis, a minimum *P* value approach was used to derive an optimal threshold for TMB as a predictive biomarker. This derived threshold was assessed as a predictive biomarker for OS benefit using a test of interaction between treatment group and TMB status in a Cox model.

### Study Oversight

The CCTG Data and Safety Monitoring Committee regularly evaluated the conduct and safety of the study. The CCTG central office performed randomization, study monitoring, and data verification. Durvalumab and tremelimumab were supplied by MedImmune/AstraZeneca.

## Results

### Patients

**Figure 1.**
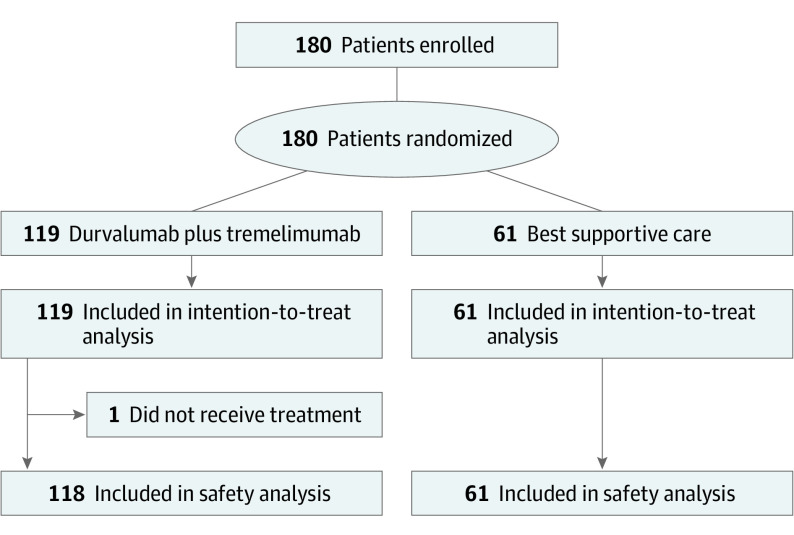
CONSORT Diagram for CCTG (Canadian Cancer Trials Group) CO.26 Study

Between August 2016 and June 2017, 180 patients were randomized, with 119 patients assigned to the treatment group and 61 patients to BSC alone. One patient did not receive durvalumab and tremelimumab as assigned; this patient was included in the treatment group for efficacy analysis but not in the safety analysis (Figure 1).

Baseline demographics are shown in Table 1. There were slightly higher proportions of female and Asian patients in the treatment group. There were no differences for presence of liver metastases or sidedness. All patients had received multiple lines of prior chemotherapy; 180 (100%) received at least 1 prior chemotherapy for advanced disease containing a fluoropyrimidine, 176 (98%) irinotecan, 154 (86%) oxaliplatin, 143 (79%) had received bevacizumab, and 47 (26%) regorafenib; 68 (97%) of patients with *RAS* wild-type tumors received cetuximab or panitumumab. No patients had received TAS-102 (trifluridine/tipiracil) during the enrollment period of this study.

**Table 1.** Baseline Characteristics of the Intention-to-Treat Population

Characteristic	No. (%)	
	Durvalumab plus tremelimumab (n = 119)	Best supportive care (n = 61)
Age, median (range), y	65 (39-87)	64 (36-85)
Sex		
Male	74 (62)	47 (77)^a^
Female	45 (38)	14 (23)
Race		
White	97 (82)	54 (89)^b^
Asian	16 (13)	3 (5)
Other	6 (5)	6 (9)
ECOG performance status		
0	33 (28)	17 (28)
1	86 (72)	44 (72)
Time from initial cancer diagnosis, median (range), mo	44 (8-181)	41 (8-152)
Presence of liver metastases		
Yes	80 (67)	47 (77)
No	39 (33)	14 (23)
Prior systemic agents		
Fluoropyrimidine	119 (100)	61 (100)
Irinotecan	118 (99)	58 (95)
Oxaliplatin	104 (87)	50 (82)
Anti-EGFR monoclonal antibody if appropriate	42 (35)	26 (43)
Bevacizumab	94 (79)	49 (80)
Regorafenib	32 (27)	15 (25)
TAS-102	0	0

Abbreviations: ECOG, Eastern Cooperative Oncology Group; EGFR, estimated glomerular filtration rate; TAS-102, trifluridine/tipiracil.

^a^ *P* = .046.

^b^ *P* = .099.

Patients in the treatment group received a median of 12 weeks of durvalumab (range, 4-84 weeks) and 12 weeks of tremelimumab (range, 4-24 weeks). Dose reduction or delay as management of adverse events was not permitted per design; however, at least 1 dose omission occurred in 50 (42.4%) patients for durvalumab and 38 (32.2%) for tremelimumab, with the most frequent reasons being investigator decision and hospitalization. In total, 102 (86.4%) and 104 (88.1%) patients received at least 90% of planned durvalumab and tremelimumab doses, respectively.

After disease progression, 8 (6.8%) and 4 (3.4%) patients in the treatment group received TAS-102 and regorafenib, whereas 3 (4.9%) patients in the BSC group received TAS-102. No patients in either groups received immune checkpoint inhibitors postprogression.

Of 180 patients enrolled, baseline blood samples were available in 169 patients (93.9%), and cfDNA analysis was successful in 168 (eTable 1 in Supplement 2). There were 2 patients with MSI-H detected in plasma, 1 in each study group. All 11 missing baseline blood samples were for patients from the BSC group, and 9 of 11 were MSS by tissue-based PCR assessment with 2 unknowns owing to a lack of tissue. There was a higher proportion of patients with *KRAS* mutation in the treatment group based on cfDNA.

### Efficacy

**Figure 2.**
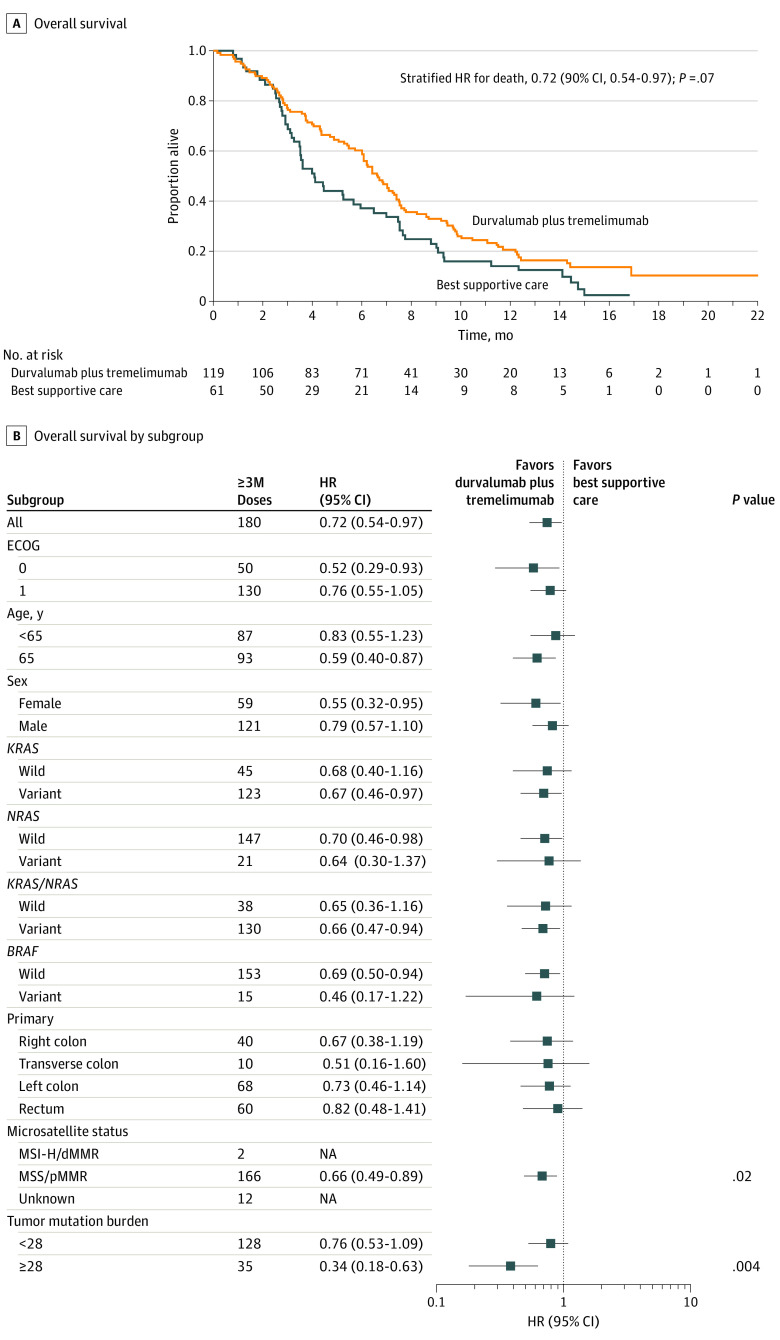
Overall Survival by Randomized Group dMMR indicates DNA mismatch repair deficient; ECOG, Eastern Cooperative Oncology Group; MSI-H, microsatellite-instability high; MSS, microsatellite stable; pMMR, DNA mismatch repair proficient. A, Overall survival; and B, overall survival by subgroups for patients treated with durvalumab and tremelimumab and best supportive care vs best supportive care alone.

At a median follow-up of 15.2 months (range, 0.16-22.0 months), 154 deaths were observed, with 149 patients (97%) dying due to disease progression. The median OS was 6.6 months for the treatment group (90% CI, 6.0-7.4 months) and 4.1 months for BSC (90% CI, 3.3-6.0 months). The HR for death was 0.72 (90% CI, 0.54-0.97; *P* = .07) (Figure 2A). In patients with MSS/pMMR tumors, the HR for death was 0.66 (90% CI, 0.48-0.89; *P* = .02) (Figure 2B).

The median PFS was 1.8 months in the treatment group (90% CI, 1.8-1.9 months), and 1.9 months in the BSC alone group (90% CI, 1.8-1.9) (eFigure 3 in Supplement 2). The HR for progression was 1.01 (90% CI, 0.76-1.34; *P* = .97).

There was no CR. One patient with MSS tumors in the treatment group had a PR that lasted longer than 21 months. Stable disease (SD) as the best response was observed in 26 (21.8%) patients of the treatment group and 4 (6.6%) patients in the BSC group. Disease control (CR, PR, or SD) was seen in 27 (22.7%) and 4 (6.6%) of patients in the treatment group and BSC, respectively (odds ratio, 4.16; 90% CI, 1.40-12.3; *P* = .006).

Plasma TMB was available for 165 patients based on baseline cfDNA. Excluding the 2 cases of MSI-H (TMB 74.7 and 247.1 vts/Mb), the median TMB was 15.3 (range, 0.96-85.4) in the treatment group and 20.9 (range, 1.9-114.9) for the BSC group, respectively (*P* = .07). Patients with TMB of 28 or more (35 of 163 MSS cases [21%]) had the greatest OS benefit (HR, 0.34; 90% CI, 0.18-0.63; *P* = .004) for the treatment group (interaction *P* = .07) (eFigures 1 and 2 in Supplement 2). In addition, high TMB was associated with a worse OS in the BSC group (HR, 2.59; 90% CI, 1.46-4.62; *P* = .007).

### Adverse Events

Adverse events were assessed in 118 patients who received at least 1 study treatment and 61 patients in the BSC group. All patients in the treatment group experienced adverse events, and 75 (62%) had at least 1 grade 3 or higher adverse event, whereas 52 (85%) of patients in the BSC group experienced adverse events, and 12 (20%) had at least 1 grade 3 or higher adverse event (*P* < .001 (Table 2). Incidences of all grades were significantly higher in the treatment group for fatigue, nausea, constipation, insomnia, cough, diarrhea, and cutaneous eruption. However, grade 3 or higher adverse events were only higher for abdominal pain (*P* = .05) and fatigue (*P* = .06).

**Table 2.** Frequency of Adverse Events and Laboratory Abnormalities^a^

Event	No. (%)			
	Durvalumab plus tremelimumab (n = 118)		Best supportive care (n = 61)	
	Any grade	Grade ≥3	Any grade	Grade ≥3
**Any event**	**118 (100)**	**75 (64)**	**52 (85)**	**12 (20)**
Most common events				
Fatigue	91 (77)	15 (13)	34 (56)	2 (3)
Anorexia	60 (51)	3 (3)	22 (36)	1 (2)
Abdominal pain	53 (45)	8 (7)	18 (30)	0
Nausea	53 (45)	0	17 (28)	0
Constipation	49 (42)	0	14 (23)	1 (2)
Dyspnea	45 (38)	6 (5)	18 (30)	2 (3)
Insomnia	43 (36)	0	11 (18)	0
Cough	41 (35)	1 (1)	10 (16)	0
Peripheral sensory neuropathy	40 (34)	0	18 (30)	0
Diarrhea	37 (31)	5 (4)	6 (10)	0
Vomiting	30 (25)	2 (2)	9 (15)	0
Macular popular eruption	28 (24)	1 (1)	5 (8)	0
Pain	27 (23)	4 (3)	9 (15)	0
Back pain	25 (21)	1	12 (20)	0
Laboratory abnormalities				
Anemia	99 (86)	15 (13)	45 (79)	3 (5)
Lymphopenia	85 (75)	26 (23	31 (55)	6 (11)
Thrombocytopenia	22 (19)	2 (1)	9 (16)	0
Leukopenia	13 (11)	4 (4)	4 (7)	0
Increase in				
Aspartate aminotransferase	69 (63)	5 (5)	33 (63)	11 (21)
Alanine aminotransferase	44 (40)	4 (4)	25 (47)	2 (4)
Total bilirubin	39 (35)	17 (13)	21 (38)	10 (18)
Alkaline phosphatase	84 (76)	19 (17)	38 (70)	14 (26)
Lactate dehydrogenase	91 (84)	20 (19)	38 (70)	8 (16)
Serum creatinine	36 (32)	2 (2)	12 (21)	2 (4)
Hypoalbuminemia	94 (85)	11 (10)	29 (55)	3 (6)
Hyponatremia	73 (64)	26 (23)	25 (46)	8 (15)
Increase in				
Amylase	13 (13)	2 (2)	6 (15)	0
Lipase	22 (22)	12 (12)	10 (24)	2 (5)

^a^ Adverse events were assessed according to *National Cancer Institute Common Terminology Criteria for Adverse Events, version 4.0*.

Incidences of all grades of laboratory abnormalities were significantly higher for lymphopenia, hypoalbuminemia, and hyponatremia in the treatment group, but there was no increase in grade 3 or higher incidences except a borderline higher incidence of lymphopenia. The incidence of TSH elevation of at least 2 × ULN (upper limit of normal) was significantly higher in the treatment group (18% vs 2%, *P* = .02), and 11 (9%) patients in the treatment group had grade 1 and/or 2 hypothyroidism compared with 1 (2%) in the BSC group. No increased incidences in other immune-mediated adverse events were observed.

Treatment with durvalumab and tremelimumab did not result in significant deterioration in physical function or global health status at 8 weeks or 16 weeks. Details of quality-of-life analysis will be reported separately.

## Discussion

One novel aspect of this study is that cfDNA analysis was successfully incorporated into a colorectal cancer study to interrogate novel predictors of immunotherapy benefit. Baseline blood samples were collected for 169 (94%) of 180 patients enrolled, and cfDNA analysis was successful in 168 of 169 patients. The median TMB in patients with MSS tumors was 16.3 (95% CI, 14.4-20.1). Further analysis revealed that the subclonal TMB (defined as those with variant allele frequency <10% of the maximal allele frequency) accounted for a substantial portion of the total TMB.^18^ The median clonal TMB was 5.8 (95% CI, 4.8-5.8), similar to the median TMB of 6 (range, 0-361) in MSS advanced colorectal cancer based on next-generation sequencing of primary tumor DNA.^19^ In a recent study, George et al^20^ reported that 424 of 1934 (21.9%) patients with MSS tumors had TMB ranging from 8.8 to 43.1. The higher TMB observed in this study may be explained by the fact that patients enrolled underwent multiple lines of systemic therapy, leading to clonal evolution and changes in the variational landscapes that may not present in the primary tumor DNA.^21^ The rate of *KRAS* and *NRAS* variation was 64% and 2% based on tissue analysis, and 78% and 10% based on cfDNA analysis (eTable 1 in Supplement 2). This hypothesis is supported by the observation that the development of resistance to EGFR inhibition is associated with downregulation of mismatch and homologous recombination repair proteins resulting in error-prone DNA repair and increased tumor variagenic ability.^22^

Tumor variation burden has emerged as a potential biomarker for response to immune checkpoint blockade. High TMB is consistently associated with benefit from immune checkpoint blockade across different types of malignant diseases, such as melanoma, lung, and bladder cancers.^21,23,24,25^ A TMB of 28 or more was found to be the optimal threshold as a potential biomarker. Patients with TMB of 28 or more receiving BSC alone had worse OS compared with those with a TMB of less than 28 receiving BSC (median OS, 3.0 vs 5.3 months; *P* = .007). As seen in eFigure 1A in Supplement 2, the HR fell gradually after a TMB of 20 until reaching a plateau. The statistical power was then gradually eroded above a TMB of 36, by which point most patients fell in the TMB less than 36 group. Similarly, the interaction *P* value stayed suppressed over an entire range of thresholds above the cut point (eFigure 1B in Supplement 2) until there were limited numbers of patients remaining in the high-TMB group, suggesting this is a real biologic phenomena and not statistical chance.

When treated with durvalumab plus tremelimumab, patients with a TMB of 28 or higher achieved significant survival improvements compared with those receiving BSC alone (median OS, 5.5 vs 3.0 months; *P* = .004). It is possible that this improvement was owing to the inferior outcome of patients receiving BSC. An interaction test showed a *P* value of .07, indicating a possible interaction between TMB and treatment with durvalumab and tremelimumab. Those with TMB of 28 or more represented 35 (21%) patients enrolled in this study. Similar benefits were observed based on clonal and subclonal TMB analysis.^18^ Samstein et al^26^ reported that the highest 20% of patients with TMB derived better OS from treatment with immune checkpoint inhibitors across different types of cancers. In colorectal cancer, a cutoff value of 52.2 was used, which was the highest among different cancers. The variable responses to immune checkpoint inhibitors in MSI-H colorectal cancer have been attributed to different variational loads among these patients.^27,28^ Peters et al^29^ recently presented blood and tissue TMB analysis from a phase 3 study of first-line durvalumab plus tremelimumab vs chemotherapy in patients with metastatic non–small cell lung cancer. Based on cfDNA, 26% patients had TMB of 20 or more. Treatment with durvalumab and tremelimumab was associated with significantly improved PFS and OS compared with chemotherapy in these patients. These data suggest that TMB is a potential biomarker for response to immune checkpoint inhibitors in advanced cancer regardless of MSI status. Genomic analysis of archival tumor tissues and serial blood samples collected while receiving treatment are ongoing.

Like other studies in advanced refractory colorectal cancer,^5,6^ PFS was short, 1.8 months in the treatment group and 1.9 months in the BSC group. Grothey et al^5^ reported a median PFS of 1.9 months in patients treated with regorafenib and 1.7 months in the placebo group. Mayer et al^6^ reported a median PFS of 2.0 months with TAS-102 and 1.7 months in the placebo group. The short PFS in this patient population and the smaller number of patients in this study (180 patients vs 760 and 800 patients in the other 2 studies, respectively) may explain why a significant benefit in OS was seen although there was no apparent improvement in PFS.

Although single-agent immune checkpoint inhibition has not shown meaningful clinical activity in MSS colorectal cancer, emerging data indicate that combining these agents with others with different mechanisms of action can potentially overcome resistance. Fukuoka et al^30^ reported a response rate of 29% in refractory MSS colorectal cancer with regorafenib and nivolumab. Combining atezolizumab with capecitabine and bevacizumab resulted in increased PFS in refractory MSS colorectal cancer.^31^ The results of this study further lend support to this strategy.

Patients in the treatment group experienced more frequent adverse events, including more frequent grade 3 or 4 adverse events. In the treatment group, 75 (64%) patients experienced at least 1 grade 3 or higher adverse event, whereas only 12 (20%) patients experienced at least 1 grade 3 or higher adverse event in the BSC group. No new safety concerns were identified. In addition, there was no significant deterioration in physical function or global health status at 8 weeks or 16 weeks.

### Limitations

All 11 patients with missing baseline blood samples were from the BSC group. Although the collection of baseline blood samples was mandated per protocol, collection was permitted after randomization but before commencement of study therapy. It is likely that these patients did not wish to undergo blood collection because of disappointments with treatment assignments. Most of these patients (9 of 11) were subsequently confirmed to be MSS based on analysis of archival tissues. The effect of these missing baseline blood samples on interpretation of our study finding is minimal.

Other important differences between study groups include higher proportions of women (*P* = .046) and Asians (*P* = .099) in the treatment group, more patients with *BRAF* variations (*P* = .13) and fewer postprogression therapies (*P* = .25) in the BSC group. These features may favor the treatment group and need to be considered when interpreting our results.

## Conclusions

This randomized phase 2 study suggests that combined PD-L1 and CTLA-4 blockade with durvalumab and tremelimumab may prolong OS in patients with heavily pretreated MSS colorectal cancer. Exploratory analysis suggests that TMB from cfDNA analysis could be a potential biomarker for benefits from immune checkpoint inhibitors. Given the lack of treatment options for this patient population, confirmation studies for combined immune checkpoint inhibitors are warranted.

## Notes

Supplement 1. **Trial Protocol.**

Supplement 2. **eTable 1.** Molecular characteristics from baseline cfDNA analysis **eFigure 1. **Hazard ratios for overall survivals (A) and the corresponding *P* value (B) with different TMB cut points **eFigure 2.** Overall Survival in patients with baseline TMB < 28 (A) and TMB ≥ 28 (B) **eFigure 3.** Progression-free survival

Supplement 3. **Data Sharing Statement.**
