# Supplementary material for: Effect of Combined Immune Checkpoint Inhibition vs Best Supportive Care Alone in Patients With Advanced Colorectal Cancer: The Canadian Cancer Trials Group CO.26 Study
Source: JAMA Oncol. 2020 May 7;6(6):1–8. doi: 10.1001/jamaoncol.2020.0910 (PMC7206536; doi:10.1001/jamaoncol.2020.0910)
Supplement: Supplement 2. — eTable 1. Molecular characteristics from baseline cfDNA analysis eFigure 1. Hazard ratios for overall survivals (A) and the corresponding P value (B) with different TMB cut points eFigure 2. Overall Survival in patients with baseline TMB < 28 (A) and TMB ≥ 28 (B) eFigure 3. Progression-free survival [file jamaoncol-6-831-s002.pdf]

Supplementary Online Content

Chen EX, Jonker DJ, Loree JM, et al. Effect of combined immune checkpoint inhibition vs best supportive care alone in patients with advanced colorectal cancer: The Canadian Cancer Trials Group CO.26 Study. *JAMA Oncol*. Published online May 7, 2020.
10.1001/jamaoncol.2020.0910

eTable 1. Molecular characteristics from baseline cfDNA analysis

eFigure 1. Hazard ratios for overall survivals (A) and the corresponding p-value (B) with different TMB cut-points

eFigure 2. Overall Survival in patients with baseline TMB < 28 (A) and TMB ≥ 28 (B)

eFigure 3. Progression-free survival

This supplementary material has been provided by the authors to give readers additional information about their work.

© 2020 Chen EX, et al. *JAMA Oncol.*

Table 1S. Molecular characteristics from baseline cfDNA analysis

Molecular Marker	Durvalumab + Tremelimumab (n = 119)	Best Supportive Care (n = 61)
**Primary site of disease – N (%)**		
**Right + transverse colon**	31 (26)	19 (31)
**Left colon + rectum**	87 (73)	41 (67)
**Unknown**	1 (1)	1 (2)
**KRAS mutation – N (%)**		
**Yes**	93 (78)	30 (49)
**No**	25 (21)	20 (33)
**Unknown**	1 (1)	11 (18)
**NRAS mutation – N (%)**		
**Yes**	11 (10)	10 (16)
**No**	107 (89)	40 (66)
**BRAF V600E mutation – N (%)**		
**Yes**	8 (7)	7 (12)
**No**	110 (92)	43 (70)
**Unknown**	1 (1)	11 (18)
**Microsatellite status – N (%)**		
**MSI-H/dMMR**	1 (1)	1 (2)
**MSS/pMMR**	117 (98)	49 (80)
**Unknown**	1 (1)	11 (18)

Abbreviations: cfDNA – cell free DNA; dMMR – deficient mismatch repair; MSI-H: microstatellite-instability high; MSS- microstatellite stable; pMMR – proficient mismatch repair

© 2020 Chen EX, et al. *JAMA Oncol.*

Figure 1S: Hazard ratios for overall survivals (A) and the corresponding p-value (B) with different TMB cut-points

A:

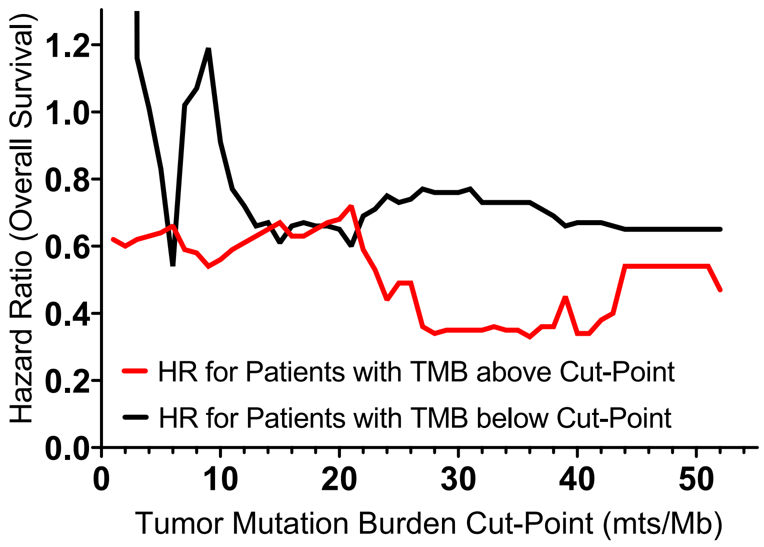

B:

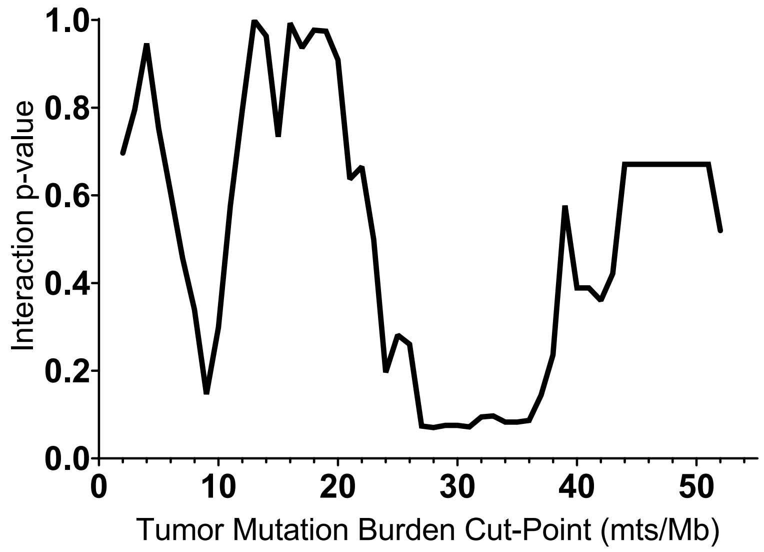

© 2020 Chen EX, et al. *JAMA Oncol.*

Figure 2S: Overall Survival in patients with baseline TMB < 28 (A) and TMB ≥ 28 (B)

A:

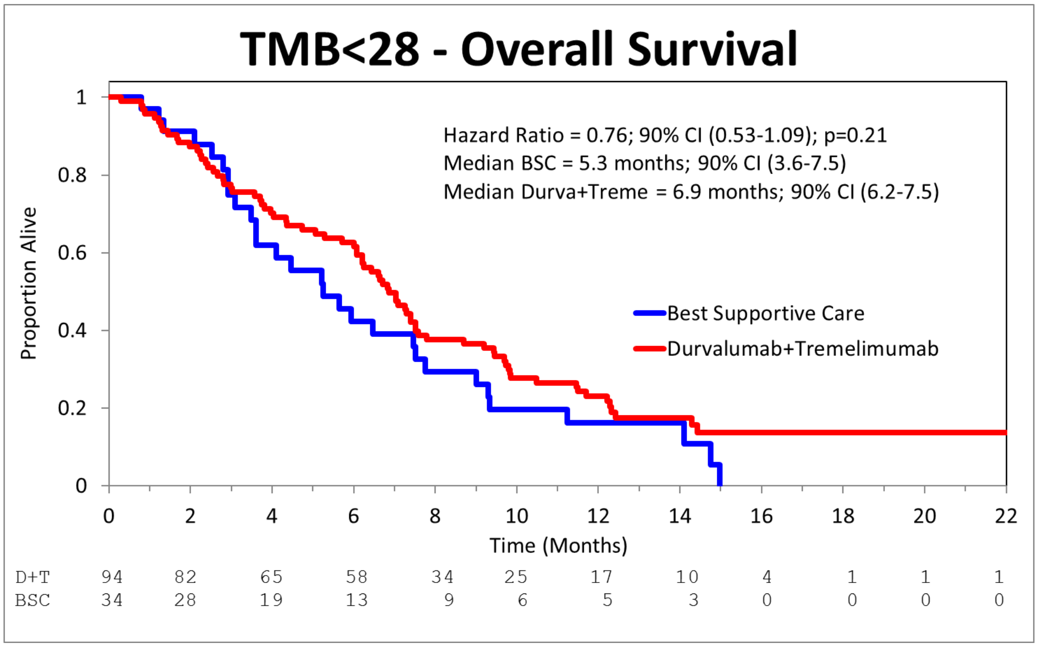

B:

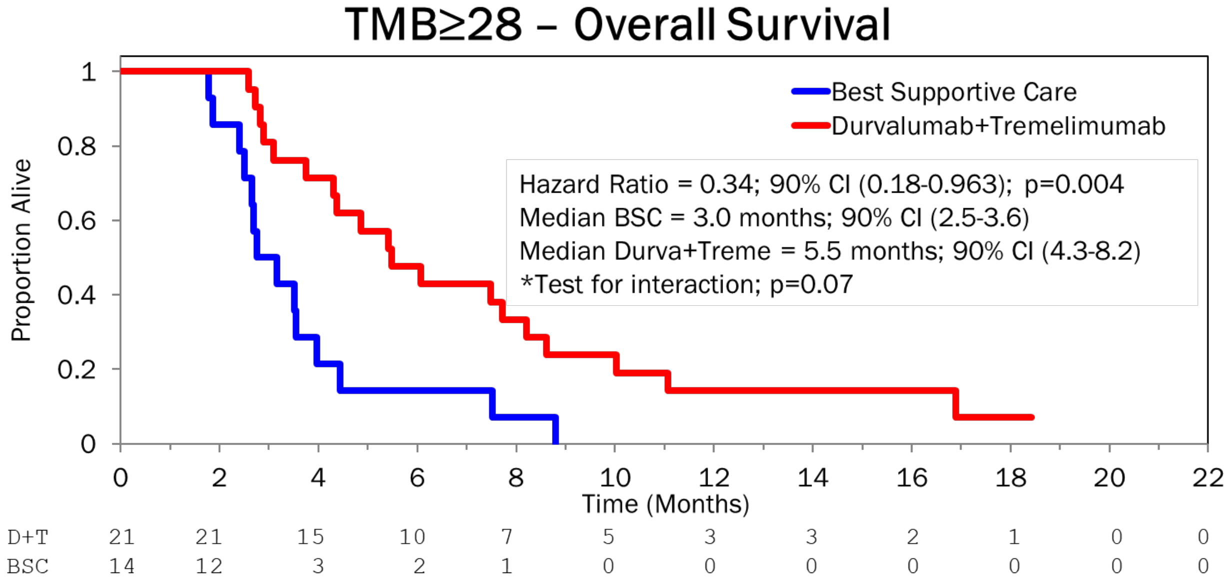

© 2020 Chen EX, et al. *JAMA Oncol.*

Figure 3S. Progression-free survival

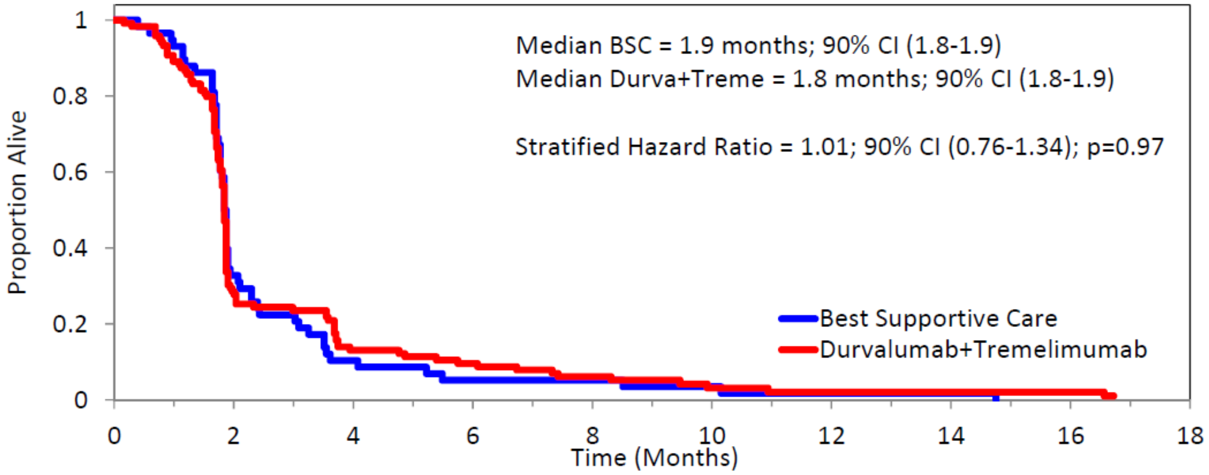

D+T	119	34	15	11	7	3	2	2	2	0
BSC	61	19	6	3	3	2	1	1	0	0

© 2020 Chen EX, et al. *JAMA Oncol.*
