# Supplementary material for: Effect of Combined Immune Checkpoint Inhibition vs Best Supportive Care Alone in Patients With Advanced Colorectal Cancer: The Canadian Cancer Trials Group CO.26 Study
Source: JAMA Oncol. 2020 May 7;6(6):1–8. doi: 10.1001/jamaoncol.2020.0910 (PMC7206536; doi:10.1001/jamaoncol.2020.0910)
Supplement: Supplement 3. — Data Sharing Statement. [file jamaoncol-6-831-s003.pdf]

Data Sharing Statement

Chen. Effect of Combined Immune Checkpoint Inhibition vs Best Supportive Care Alone in Patients With Advanced Colorectal Cancer. *JAMA Oncol*. Published May 07, 2020. 10.1001/jamaoncol.2020.0910

Data
Data available: Yes
Data types: Deidentified participant data
How to access data: Data collected from this study, including individual participant data and a data dictionary defining each field, will be made available to interested researchers. The Canadian Cancer Trials Group (CCTG) has an established request procedure and interested investigators should submit a brief proposal using the Request for Data Proposal Form available at http://www.ctg.queensu.ca/. Upon approval, de-identified individual participant data, and relevant study documents (protocol and statistical analysis plan) will be made available.
When available: With publication

Supporting Documents
Document types: None

Additional Information
Who can access the data: Data collected from this study, including individual participant data and a data dictionary defining each field, will be made available to interested researchers. The Canadian Cancer Trials Group (CCTG) has an established request procedure and interested investigators should submit a brief proposal using the Request for Data Proposal Form available at http://www.ctg.queensu.ca/. Upon approval, de-identified individual participant data, and relevant study documents (protocol and statistical analysis plan) will be made available.
Types of analyses: Data collected from this study, including individual participant data and a data dictionary defining each field, will be made available to interested researchers. The Canadian Cancer Trials Group (CCTG) has an established request procedure and interested investigators should submit a brief proposal using the Request for Data Proposal Form available at http://www.ctg.queensu.ca/. Upon approval, de-identified individual participant data, and relevant study documents (protocol and statistical analysis plan) will be made available.
Mechanisms of data availability: Data collected from this study,

including individual participant data and a data dictionary defining each field, will be made available to interested researchers. The Canadian Cancer Trials Group (CCTG) has an established request procedure and interested investigators should submit a brief proposal using the Request for Data Proposal Form available at http://www.ctg.queensu.ca/. Upon approval, de-identified individual participant data, and relevant study documents (protocol and statistical analysis plan) will be made available.
